# Infected Urachal Cyst Presenting as a Huge Abdominal Abscess in an Elderly: A Case Report

**DOI:** 10.7759/cureus.115228

**Published:** 2026-08-26

**Authors:** Mohamed Belmejjad, Kamal Mohamed, Yassine Karmouch, Kamal Moufid, Fouad Hajji

**Affiliations:** 1 Department of Urology, Ibn Sina Military Hospital, Cadi Ayyad University, Marrakesh, MAR

**Keywords:** adult urachal cyst, infected urachal cyst, percutaneous drainage, urachal abscess, urachal anomalies

## Abstract

Urachal anomalies are rare congenital disorders in which an embryonic vestige of the allantois persists after birth as a tubular remnant. These urachal remnants may present with various clinical conditions that are mainly detected during infancy and childhood but are exceedingly rare in adulthood. We report a rare case of a 63-year-old man with a history of recurrent purulent umbilical discharge who presented with painful swelling and erythema of the periumbilical area, accompanied by fever, suggesting an intra-abdominal or abdominal wall abscess on physical examination. Abdominal computed tomography and magnetic resonance imaging revealed a large urachal cyst abscess. As the patient required repeated percutaneous drainage procedures because of abscess recurrence, radical en bloc excision of the urachal remnant was subsequently performed via an open approach, with good outcomes. This case emphasizes the clinical importance of considering complicated urachal disorders, such as infected urachal cysts, in the differential diagnosis of acute abdominal pain in adults, as well as the role of primary percutaneous drainage in the initial management of large abscesses. We also highlight the need for subsequent complete excision of the urachal remnant, which may help prevent recurrent infections and abscess formation and reduce the potential risk of malignant transformation, particularly in adulthood.

## Introduction

The urachus is an embryonic vestige of the allantois that runs preperitoneally between the urinary bladder and the umbilicus [1,2]. This tubular structure is completely obliterated at birth to form a fibromuscular cord, the median umbilical ligament [3]. Failure of this involution is a rare congenital anomaly that results in a urachal remnant, which persists in approximately 1 in 5,000 newborns [1]. However, the precise overall incidence of urachal anomalies in adults is unknown because these conditions are very rare in adulthood, are frequently misdiagnosed, and have been documented almost exclusively in isolated case reports and small clinical series.

Four main types of congenital urachal disorders are recognized, most of which are diagnosed during infancy and childhood: patent urachus (approximately 50%), urachal cyst (approximately 30%), umbilical-urachal sinus (approximately 15%), and vesico-urachal diverticulum (approximately 3%-5%) [1-3]. Most cases are asymptomatic or present with nonspecific complaints such as umbilical discharge, abdominal pain or a palpable mass, dysuria, recurrent urinary tract infections, and fever [1-3]. They are often detected incidentally during imaging or surgery for unrelated symptoms or may remain undiagnosed until complications, such as infection and/or malignant transformation, occur [2,3].

An infected urachal cyst presenting as an abdominal abscess is an uncommon entity with nonspecific clinical features that may overlap with those of various other conditions, particularly in elderly patients, who frequently have atypical presentations and multiple coexisting chronic comorbidities. This poses both diagnostic and therapeutic challenges.

Herein, we discuss the case of a 63-year-old man who presented with a painful swelling and erythema in the periumbilical area, accompanied by fever, and was found to have a huge urachal cyst abscess, an exceedingly rare clinical presentation.

This case is particularly noteworthy because of the delayed diagnosis of a congenital urachal cyst that initially manifested as recurrent purulent umbilical discharge, followed by rapid recurrence of a urachal abscess after conservative, nonoperative management.

## Case presentation

A 63-year-old man presented to the emergency department with a two-week history of progressive painful swelling and erythema of the periumbilical area, decreased appetite, weight loss, and fever. He denied any gastrointestinal or genitourinary complaints. Three years earlier, he had experienced two episodes of purulent umbilical discharge, which were successfully treated with empirical antibiotics prescribed by his general practitioner.

**Figure 1:**
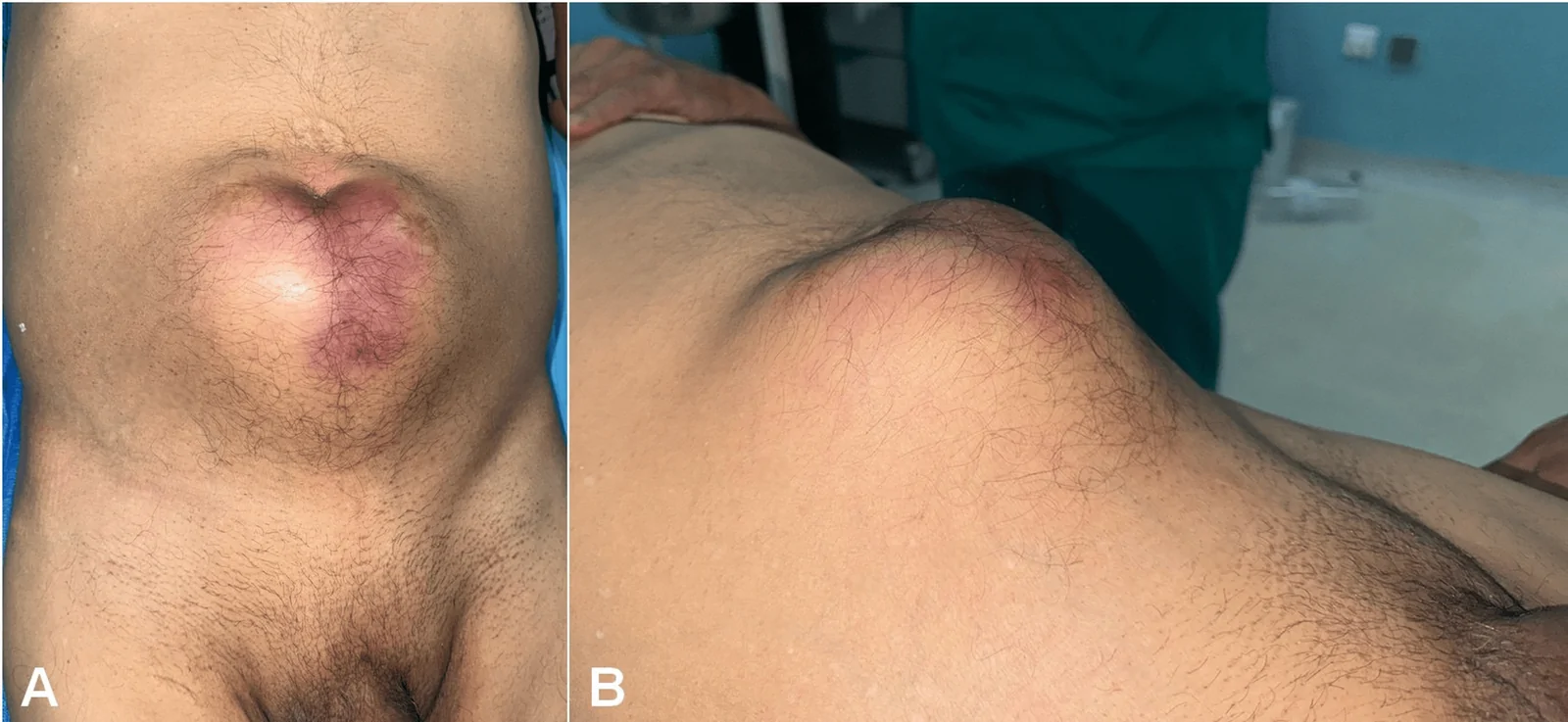
Physical examination revealing a periumbilical tender, erythematous, warm, and fluctuant mass (A) Front view. (B) Lateral view.

His vital signs were stable, except for an elevated temperature of 39°C. Physical examination revealed a tender, erythematous, warm, and fluctuant periumbilical mass (Figure 1). Given his history of recurrent purulent umbilical discharge, the palpable mass along the infraumbilical midline, and the periumbilical erythema, an infected urachal remnant was considered the most likely diagnosis. However, the differential diagnosis included a primary abdominal wall abscess; a neoplasm (e.g., advanced colonic malignancy or lymphoma) with central necrosis and superinfection; an incarcerated or strangulated hernia; a rectus sheath hematoma; and an intra-abdominal abscess resulting from severe complications of colonic diverticulitis, Crohn's disease, or appendicitis.

Laboratory tests revealed an elevated C-reactive protein level of 319.63 mg/L (normal, <5 mg/L), an elevated procalcitonin level of 6.4 ng/mL (normal, <0.5 ng/mL), hyperleukocytosis with a white blood cell count of 20.7 × 10³/µL (normal range, 4.0-11.0 × 10³/µL), and a neutrophil count of 17.4 × 10³/µL (normal range, 1.4-7.7 × 10³/µL).

**Figure 2:**
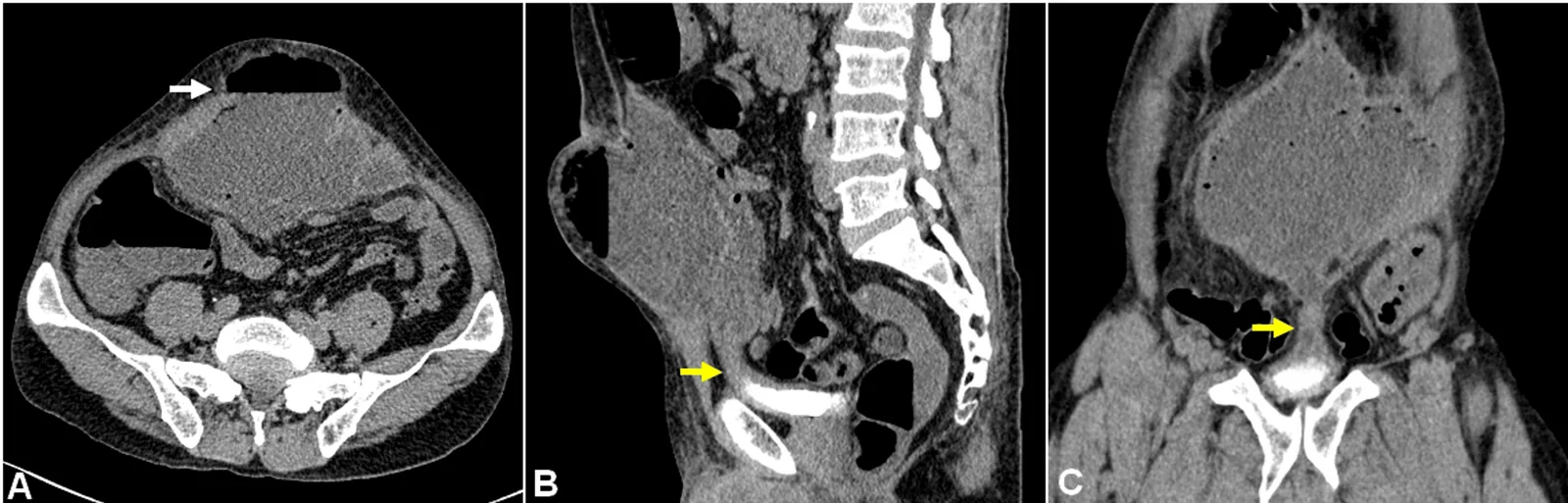
Abdominal contrast-enhanced CT scan image showing a large urachal abscess (A) Axial image showing a thick-walled heterogeneous cystic mass in the preperitoneal space containing a gas-fluid level (white arrow). (B) Coronal and (C) sagittal images showing a preperitoneal cystic mass below the umbilicus, with a cord-like structure that extends to the bladder (yellow arrows), but no evidence of communication between the mass and the bladder.

**Figure 3:**
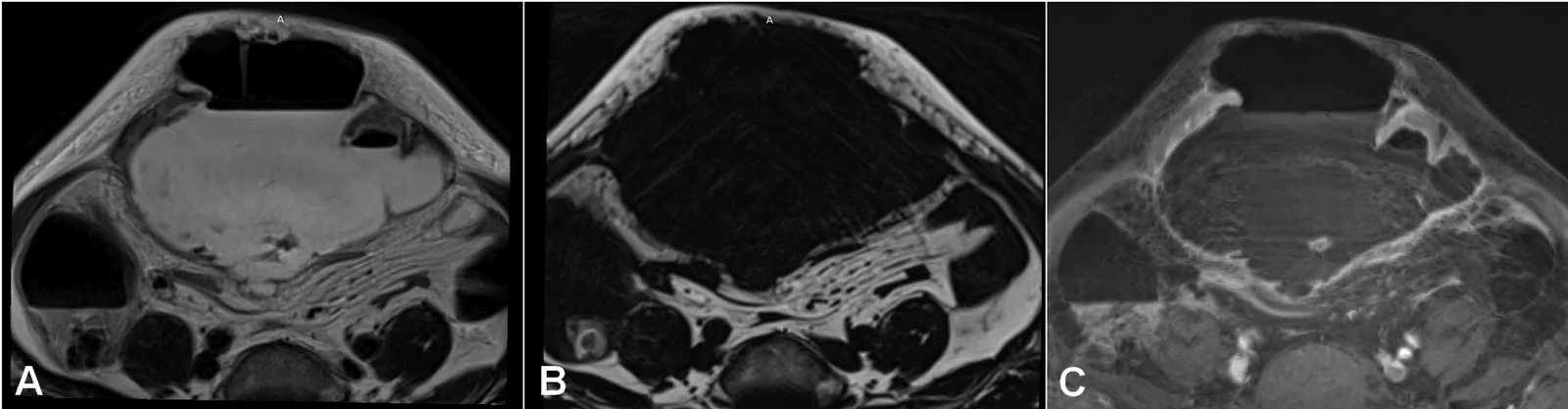
Axial magnetic resonance images showing a large urachal abscess with no signs of malignant degeneration The abscess is seen as a heterogeneous hyperintense signal on T2-weighted image (A), and as a hypointense signal on T1-weighted image (B), with peripheral contrast enhancement (C).

Contrast-enhanced abdominal computed tomography (CT) demonstrated a 14 × 13 × 10 cm thick-walled, heterogeneous cystic mass in the preperitoneal space of the lower abdominal wall below the umbilicus, containing a gas-fluid level, with a 3.6-cm cord-like structure extending to the anterosuperior aspect of the bladder. There was no evidence of communication between the mass and the bladder (Figure 2). An abscess arising from a urachal cyst was considered the most likely diagnosis. Magnetic resonance imaging (MRI) of the abdomen confirmed these findings and showed no evidence of malignancy (Figure 3).

**Figure 4:**
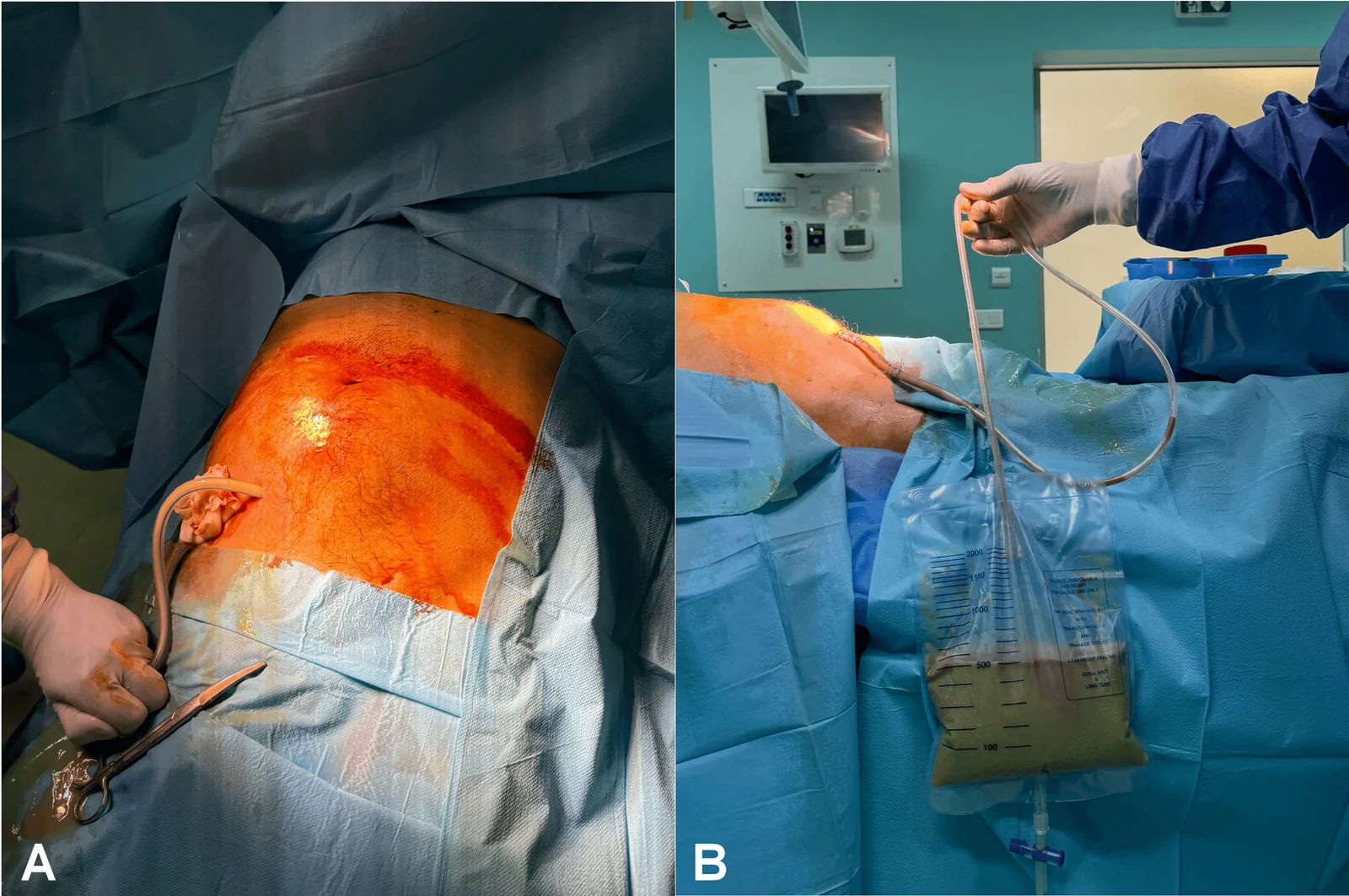
Intraoperative view showing drainage of the abscess via a percutaneous approach (A) A 28-French thoracic catheter was inserted percutaneously. (B) Approximately 600 mL of thick pus was drained from the abscess.

The patient was promptly admitted for percutaneous drainage, and ceftriaxone and gentamicin were administered empirically as intravenous antibiotics. A 600 mL volume of thick, purulent, and malodorous material was obtained (Figure 4).

The patient’s symptoms improved dramatically, accompanied by marked biochemical improvement. The aspirated pus culture grew *Escherichia coli*, while cytological examination showed no malignant cells. Urine and blood cultures showed no microbial growth. The drain was removed because of low output after a follow-up CT scan, and the patient was discharged home on the seventh day with oral antibiotics.

**Figure 5:**
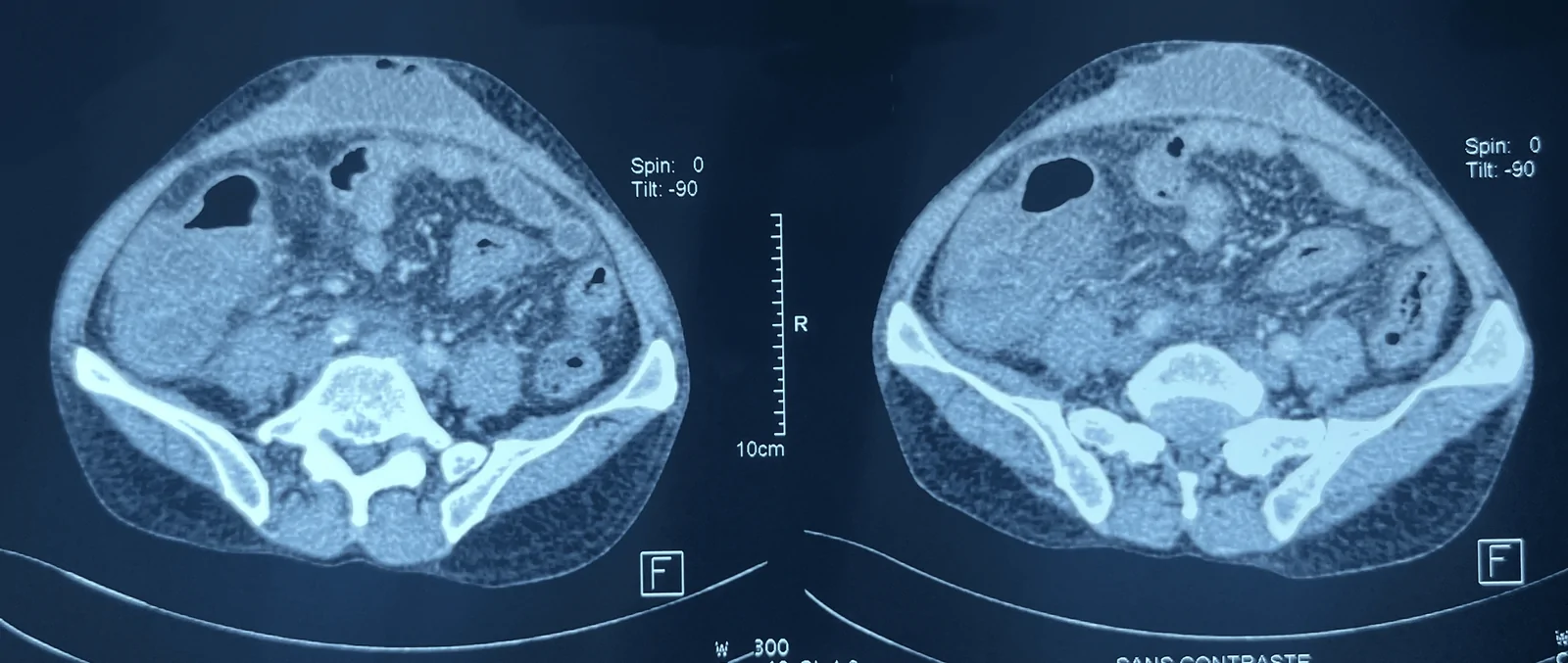
Axial abdominal contrast-enhanced CT scan image showing a recurrent urachal abscess

Two weeks later, he again developed the same symptoms, and the CT demonstrated a 6 × 2 cm abscess below the umbilicus (Figure 5), which was successfully drained using a pigtail catheter. *E. coli* was again isolated, and antibiotic therapy was adjusted according to susceptibility testing. Cystoscopic examination showed no evidence of malignancy or communication with the abscess cavity.

**Figure 6:**
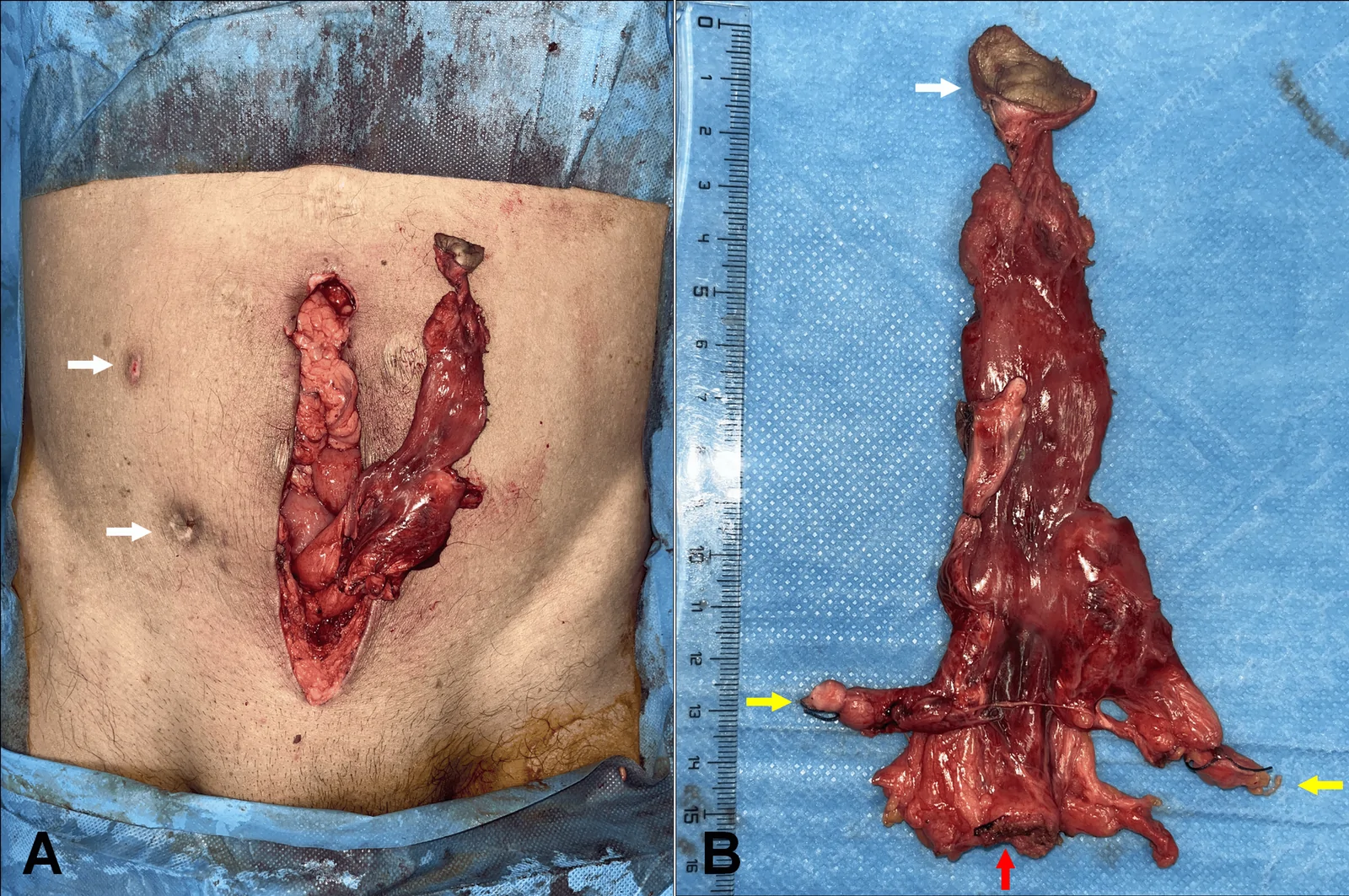
Intraoperative view showing radical en bloc excision of the urachal remnant (A) Open midline infra-umbilical approach and scars left by both drainage catheters (white arrows). (B) Gross specimen of the urachal remnant, including the umbilicus (white arrow), both medial umbilical ligaments (yellow arrows), and a bladder cuff (red arrow).

The patient subsequently underwent surgery because of the recurrence. The urachal remnant was excised through an open midline infraumbilical approach (Figure 6). The postoperative course was uneventful.

**Figure 7:**
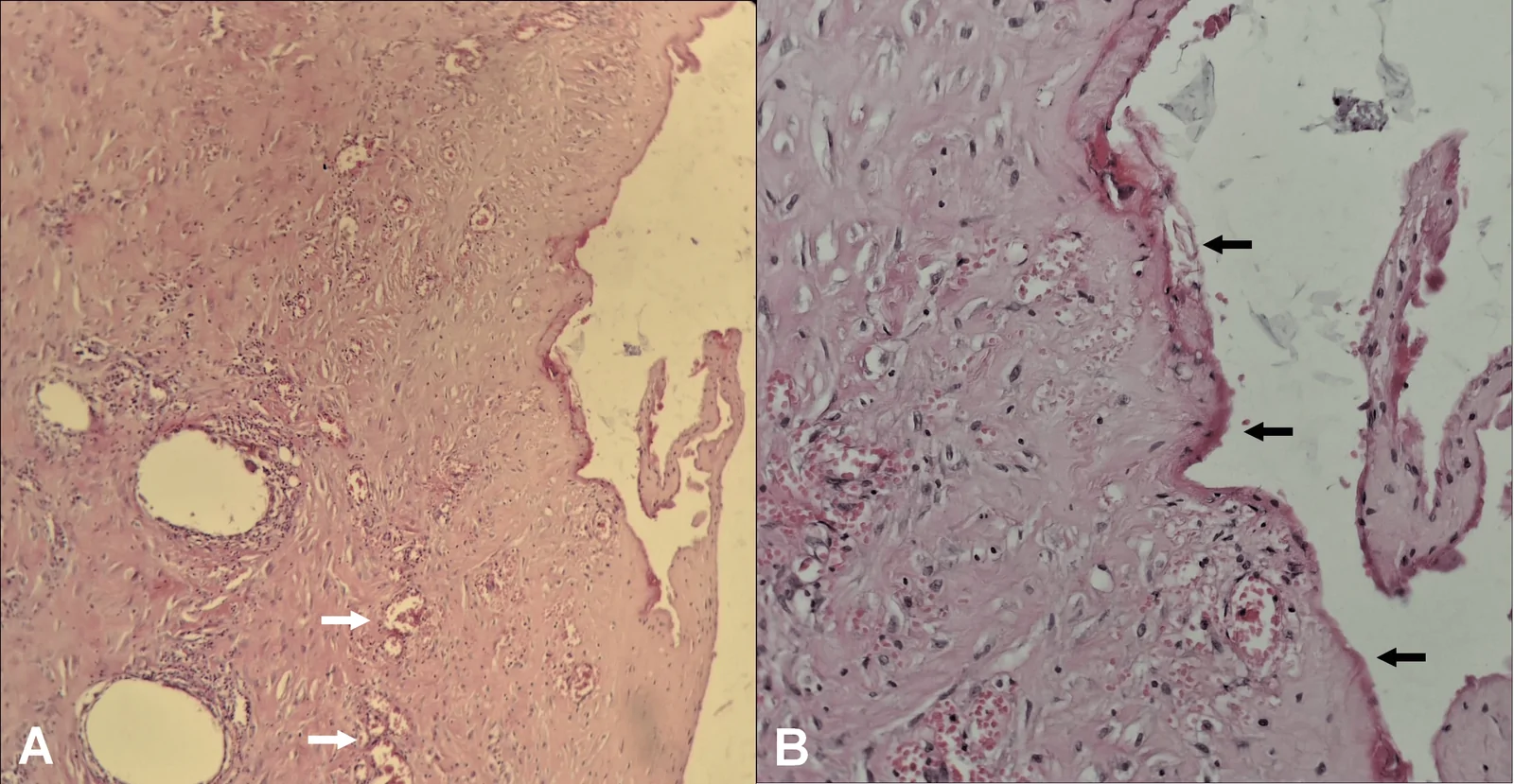
Photomicrograph image showing histopathological features of an epithelial-lined urachal cyst wall (A) Original magnification ×60. (B) Original magnification ×100. Hematoxylin and eosin staining (H&E) showing dense fibrous tissue with a polymorphous inflammatory infiltrate associated with vascular congestion (white arrows), lined by a flattened and partially ulcerated epithelium that is composed of a few regular cuboidal epithelial cells without cytological atypia (black arrows).

Pathological examination of the urachal cyst wall confirmed pressure-induced atrophy of the lining epithelium and chronic inflammation of the surrounding tissue, with no evidence of malignancy (Figure 7). At 18 months of follow-up, the patient showed no evidence of recurrence or malignancy.

## Discussion

The true incidence of congenital urachal disorders appears to be underestimated, but these disorders show a marked pediatric predominance [2,4]. In this setting, umbilical discharge is the most frequent presenting complaint, followed by abdominal pain and a palpable mass [4].

However, umbilical discharge is an uncommon clinical complaint encountered in adult primary care settings and has a broad differential diagnosis. While abdominal wall pathologies, such as acquired umbilical conditions, are frequently considered [5,6], intra-abdominal or visceral pathologies are often overlooked, leading to diagnostic delays. Among these, urachal anomalies are rare congenital disorders that may go unnoticed or may not be appropriately managed by clinicians who are unaware of the potential risks of recurrent and/or severe infection, as well as malignant transformation, particularly in adulthood [1-4]. Hence, in adults presenting with persistent or recurrent umbilical discharge, urachal anomalies should be considered in the differential diagnosis. We present a rare case of a late diagnosis of an infected urachal cyst that led to the formation of a huge intra-abdominal abscess in an older man whose medical history revealed recurrent episodes of umbilical discharge.

Pathways of urachal cyst infection may be hematogenous, lymphatic, or from the adjacent urinary bladder [1,2]. We are uncertain about the origin of the infection in our patient; there was no communication with the urinary bladder, no associated adjacent or distant occult deep-seated infection, and no similar microorganisms were isolated from urine or blood cultures. However, it seems reasonable to associate the infected urachal cyst with an intestinal origin because of the presence of enterobacteria (i.e., *E. coli*). Accordingly, we assumed that bacterial translocation from the intestine had occurred, causing infection of the urachal cyst, most likely via a lymphatic route.

Infected urachal anomalies have nonspecific clinical findings and can mimic other abdominal and pelvic inflammatory disorders. Physical examination, medical history, and laboratory findings are not specific for the diagnosis of these congenital anomalies. In the current case, the condition could have been easily misdiagnosed as an abdominal wall or intraperitoneal abscess based solely on the physical examination. However, the patient had no predisposing risk factors, such as prior trauma or surgery, underlying infection or malignant tumors, systemic disease, or an immunocompromised state [7]. Hence, clinicians have to rely on imaging to establish the diagnosis.

Urachal cyst abscesses may be readily diagnosed by ultrasound [2,8,9]. In the current case, identification of a cord-like structure connecting the abscess to the bladder dome on sagittal CT was a key diagnostic feature. Although some cases may mimic malignant tumors on ultrasound and CT [8,9], MRI may provide additional information, particularly to exclude unrelated malignancies or better assess associated urachal neoplasms [10]. Besides the cystoscopic evaluation performed in our patient, cystography may also be a helpful diagnostic tool, particularly for ruling out communication with the urinary bladder, especially in the setting of recurrent urachal abscesses.

If left untreated, an infected urachal cyst may lead to abscess formation, as in this case, as well as fistula formation, rupture, sepsis, and even death [2,11].

There are currently no guidelines for the management of urachal cyst abscesses. In addition to appropriate antibiotics, treatment may include percutaneous or open surgical drainage and excision of the infected urachal remnant through an open, laparoscopic, or robotic approach [12,13]. The present case could initially have been managed conservatively; percutaneous drainage alone appeared feasible, safe, and effective. However, potential risks include infection recurrence and abscess recurrence (approximately 30% reinfection rate) [2], as occurred in our patient. Furthermore, the benefits of radical surgery outweighed the potential risk of tumor development years later, particularly in the presence of an epithelialized remnant exposed to prolonged inflammation [14]. Indeed, epithelial components and long-term exposure to infection and inflammation, as seen in this case, have both been postulated as risk factors for urachal malignancy later in life, most commonly adenocarcinoma (94%) [4]. However, single-stage resection of a large infected urachal cyst could result in higher postoperative complication rates and longer hospital stays [1]. With this in mind, percutaneous drainage with culture-guided antibiotic therapy followed by radical en bloc urachectomy with omphalectomy and partial cystectomy represents a reasonable treatment option for this condition.

Finally, had the recurrent umbilical discharge been appropriately investigated in this older man, earlier recognition of an uncomplicated urachal cyst could have led to achievable, efficient, and cost-effective management through single-stage laparoscopic removal of the urachal remnant [15].

## Conclusions

Although urachal cyst abscess is uncommon in adulthood, it should be considered in the differential diagnosis of acute periumbilical and lower abdominal pain, particularly in the presence of a midline mass, periumbilical erythema, and a history of recurrent umbilical discharge. Primary percutaneous drainage of the urachal cyst abscess, followed by culture-guided antibiotic therapy and radical en bloc excision of the urachal cyst, is a safe and effective treatment option that may help prevent recurrent infection and abscess formation, as well as reduce the potential risk of malignant transformation.
